# Neonicotinoid Levels Exceed EPA Benchmarks in Half of Waterways on Illinois Protected Lands

**DOI:** 10.1007/s00128-026-04311-1

**Published:** 2026-08-20

**Authors:** Tara C. Hohoff, Erika E. Bilger, Ricky Gieser, Mark A. Davis

**Affiliations:** 1 University of Illinois Urbana-Champaign, Prairie Research Institute, Champaign, IL USA; 2 School of Exercise Science and Sport, Millikin University, Decatur, IL USA

**Keywords:** Contaminants, Public lands, Insects, Agriculture

## Abstract

Neonicotinoids have become widely used pesticides due to their specificity for insects. We explored how these contaminants are distributed on protected lands within Illinois. We hypothesized that there would be lower insect biomass at sites with higher neonicotinoid concentrations which we expected to occur at sites with a higher surrounding proportion of agriculture. We predicted that neonicotinoid levels would be highest at the start of the season, coincident with planting, then taper off throughout the growing season. Our results revealed that 25 of 50 sites surveyed had concentrations of at least one neonicotinoid (imidacloprid or clothianidin) over United States Environmental Protection Agency (EPA) benchmarks for chronic exposure. Imidacloprid had high detection above EPA benchmarks in Cook County while clothianidin was more often above benchmarks in central Illinois. There was a negative relationship between clothianidin levels and ratio of agriculture on measured insect biomass at sites with a higher proportion of agriculture. We did not identify a clear peak of imidacloprid or clothianidin concentrations. This study suggests the high level of contamination found at protected sites is concerning for ecosystem health as insects are the target prey for many wildlife species.

## Introduction

Neonicotinoids are commonly used both commercially and domestically due to their specificity for insects and minimal direct impact to mammal species. These insecticides bind to nicotinic acetylcholine receptors, which are more abundant in insects, disrupting nerve function, causing paralysis and death (Jeschke and Nauen 2008). Neonicotinoids are mostly applied using soil or seed treatments but are also sprayed on crops (Jeschke et al. 2011). These chemicals are found in the pollen and nectar of treated vegetation (Wood and Goulson 2017). Only up to 20% of the active ingredient in neonicotinoids is absorbed by vegetation, with the remainder entering the soil (Sur and Stork 2003). These compounds have wide-ranging half-lives in soil, typically from 200 to 1000 days, which means repeat applications can accumulate over time (Goulson 2013). Neonicotinoids are likely mobilized via rain events and transported into waterways via tile drainage systems (Chrétien et al. 2017). Consequently, neonicotinoids are pervasive in surface water, with clothianidin and imidacloprid positively related to nearby land-use (Hladik and Kolpin 2015; Hladik et al. 2018).

A meta-analysis by Main et al. (2018) found that neonicotinoids have a negative impact on behavior and survival of non-target terrestrial arthropods including 6 guilds: detritivores, mixed, omnivores, parasitoids, pollinators, and predators. These terrestrial insects may ingest neonicotinoids through consumption of vegetation, pollen, nectar or contaminated insects.

Vertebrate wildlife is affected directly, such as the ingestion of treated seeds or contaminated prey insects, or indirectly, through reduction of prey for insectivores (Baxter et al. 2005; Gibbons et al. 2014; Hallman et al. 2014). For example, a 2022 study of big brown bat specimens from throughout Missouri revealed that eight commonly used pesticides, three of which are neonicotinoids (clothianidin, imidacloprid, and thiamethoxam), had bioaccumulated in the bats (Hooper et al. 2022). Neonicotinoid residue has also been detected in European honey buzzard blood samples whose main prey are pollinating bees and wasps (Byholm et al. 2018). The cascading impact of neonicotinoids on ecosystems, including non-target invertebrates and vertebrate wildlife is likely widespread and significant (Sánchez-Bayo and Goka 2016).

We tested the hypothesis that the concentration of neonicotinoids in Illinois waterbodies would impact local insect populations with aquatic levels indicating nearby exposure levels. Since there is extensive research on the influence of neonicotinoids on daytime insects (Pisa et al. 2015), we used nighttime insect sampling to understand how they may be impacted. We expected the landscape context to influence neonicotinoid concentration since riparian buffers or protected lands may aid in reducing pesticide loads in aquatic systems (Reichenberger et al. 2007; Bereswill et al. 2014). We tested 50 sites throughout the state of Illinois, the landscape of which ranges from highly urban in Chicagoland, to intensive agriculture in central Illinois and a large portion of southern Illinois protected as the Shawnee National Forest. We hypothesized that central Illinois, where intensive row-crop agriculture dominates the landscape, would have the highest detection rate of neonicotinoids and lowest arthropod density. We also expected that there would be trends in the neonicotinoid concentrations by date due to time of application with the highest concentrations occurring at the beginning of the survey season closest to seed planting and concomitant with spring rainfall runoff.

## Methods

For selection of survey sites, we used the “protected lands” geodatabase accessible from the Prairie State Conservation Coalition (https://prairiestateconservation.org; via I-View) and used the designation of “public lands,” which includes preserves, parks and county owned facilities. To identify water sources, we used the National Hydrography Dataset area features (https://www.usgs.gov/national-hydrography/national-hydrography-dataset). Once the shapefiles were imported into ArcMap 10.8.1, we generated 60 sites within 50 m of water on public land using the Spatially Balanced Points tool. Due to permitting and access, we were only able to collect samples from 50 sites, which ranged from Shawnee National Forest to county owned golf courses near the Chicagoland area, with water bodies including streams, rivers, lakes, wetlands, and a canal. We collected samples from 19 May 2021 to 16 August 2021. At each site we collected one water sample from open water as close to the generated point depending on accessibility using a 60-milliliter syringe to fill a 275-milliliter amber glass jar. We stored samples on ice in a cooler until transferred to a 40 °C refrigerator. Samples were mailed in coolers with ice to the University of Nebraska Water Sciences Laboratory for analysis of 21 neonicotinoids (Table 1) within 30 days of collection. Samples were analyzed using liquid chromatography/mass spectrometry following Maclean et al. 2025. To assess potential risks, we compared our measured concentrations to the freshwater invertebrate chronic Aquatic Life Benchmarks (USEPA 2021).

**Table 1:** Neonicotinoids and related chemicals tested from 50 sites in Illinois by the University of Nebraska Water Sciences Laboratory and associated United States Environmental Protection Agency benchmarks (U.S. EPA 2021). Reporting limit is the lowest concentration that could be measured by UNLWS

Analyte	Chemical class	Compound type	Reporting limit (µg L^−1^)	Number of detects (> chronic benchmark)	EPA acute benchmark (µg L^−1^)	EPA chronic benchmark (µg L^−1^)
Acetamiprid	Neonicotinoid insecticide	Parent compound	0.009	0	10.50	2.10
Azoxystrobin	Fungicide	Parent compound	0.030	0	130.00	44.00
Clothianidin	Neonicotinoid insecticide	Parent compound	0.009	13	11.00	0.05
Dimethoate	Insecticide / acaricide	Parent compound	0.008	0		
Dinotefuran	Neonicotinoid insecticide	Parent compound	0.007	0	484,150.00	95,300.00
Imidacloprid	Neonicotinoid insecticide	Parent compound	0.010	22	0.39	0.01
Indoxacarb	Insecticide	Parent compound	0.005	0	300.00	75.00
Metalaxyl	Fungicide	Parent compound	0.008	0	14,000.00	1,200.00
Picoxystrobin	Fungicide	Parent compound	0.013	0	12.00	1.00
Pyraclostrobin	Fungicide	Parent compound	0.006	0		
Thiacloprid	Neonicotinoid insecticide	Parent compound	0.017	0	18.90	0.97
Thiamethoxam	Neonicotinoid insecticide	Parent compound	0.011	0	17.50	0.74
Trifloxystrobin	Fungicide	Parent compound	0.009	0	12.65	2.76
6-Chloronicotinic acid	Neonicotinoid metabolite	Transformation product	0.063	0		
6-Chloronicotinic aldehyde	Neonicotinoid metabolite	Transformation product	0.013	0		
6-Chloro-N-methylnicotinamide	Neonicotinoid metabolite	Transformation product	0.007	0		
Imidacloprid desnitro	Neonicotinoid metabolite	Transformation product	0.023	0	28,000.00	28,000.00
Imidacloprid olefin	Neonicotinoid metabolite	Transformation product	0.013	0		
Imidacloprid urea	Neonicotinoid metabolite	Transformation product	0.024	0	47,400.00	47,400.00
Thiamethoxam urea	Neonicotinoid metabolite	Transformation product	0.005	0		
Sulfoxaflor	Sulfoximine insecticide	Neonicotinoid-like	0.009	0	7.85	4.00

At each site we also collected insects using a BioQuip bucket trap on the ground loaded with ethyl acetate (CAS#141-78-6, University of Illinois School of Chemical Sciences Storeroom) for two nights starting on the day of the water collection. The light was controlled by a timer which turned the light on at sunset and turned it off three hours later. Insects were collected each morning and preserved on ice until stored in a freezer. The samples were thawed for 4 hours in a chemical fume hood, then dried for five days in either a Fisher Isotemp 500 Series or Blue M 0–12 A drying oven before weighing for biomass.

To estimate the amount of agriculture surrounding survey locations, we used CropScape (USDA 2021) land cover extracted from HUC12 Watershed Boundaries (NRCS 2005) in ArcGIS Pro 3.6.0. We calculated the proportion of agriculture in the watershed by summing the total area designated as crops and dividing by the total area in the watershed. We focused statistical analysis on clothianidin since this is more commonly associated with agriculture. We analyzed concentrations on emergent insects and the agricultural context using RStudio (2026.01.0; R version 4.5.2). Insect biomass was analyzed using generalized linear models (GLM) with a Gamma distribution and a log link function (R package glm2; Marschner 2018). The Gamma family was selected because insect biomass measurements were positive and right‑skewed. We tested a series of models and compared them using Akaike information criterion (Burnham and Anderson 2002). SiteID was not included in final models because it had a zero-variance estimate indicating that it was not adding to the model fit.

## Results

**Fig. 1:**
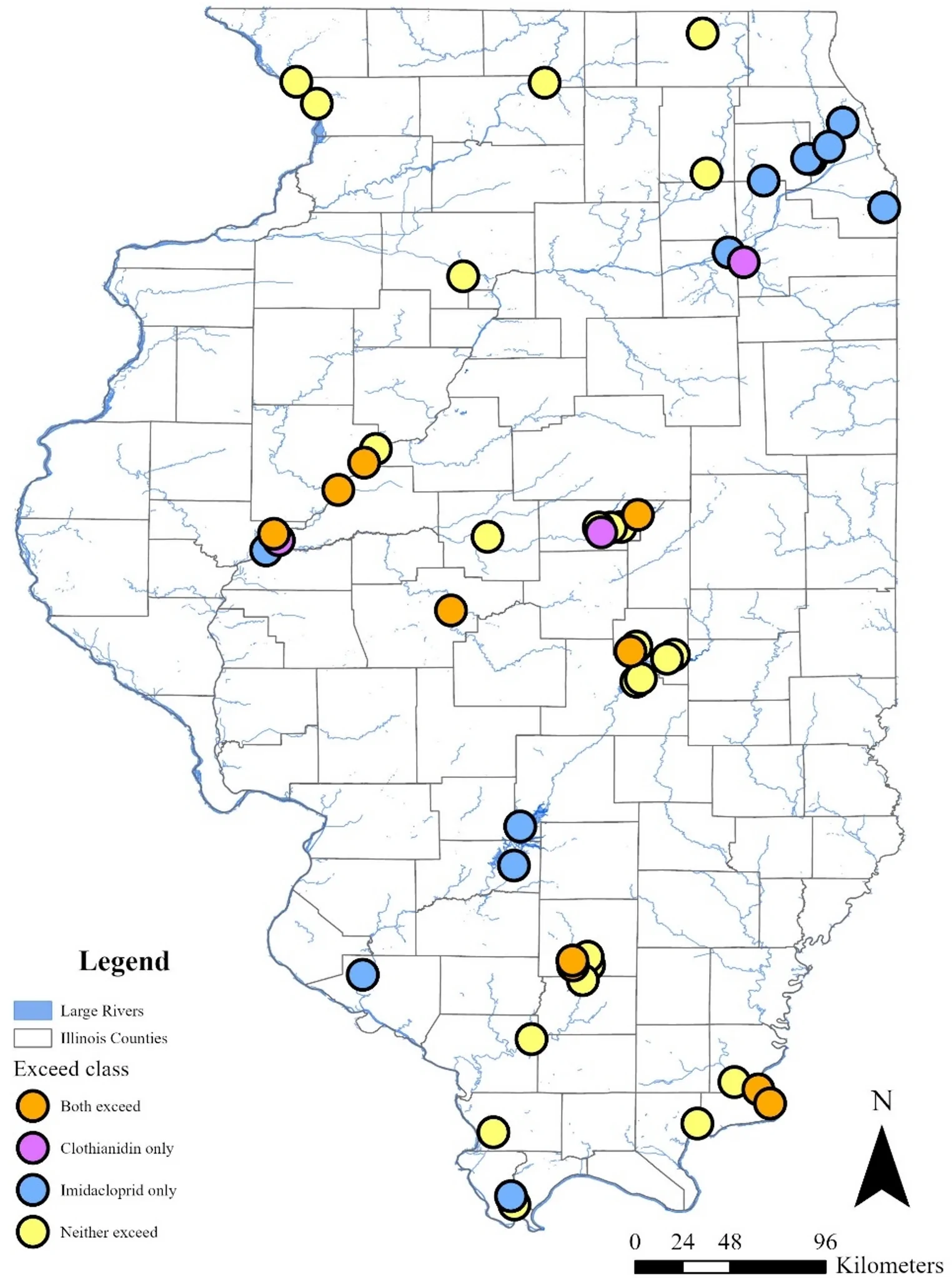
Site locations where neonicotinoid levels for clothianidin and imidacloprid are above EPA benchmarks for chronic exposure to freshwater invertebrates

**Fig. 2:**
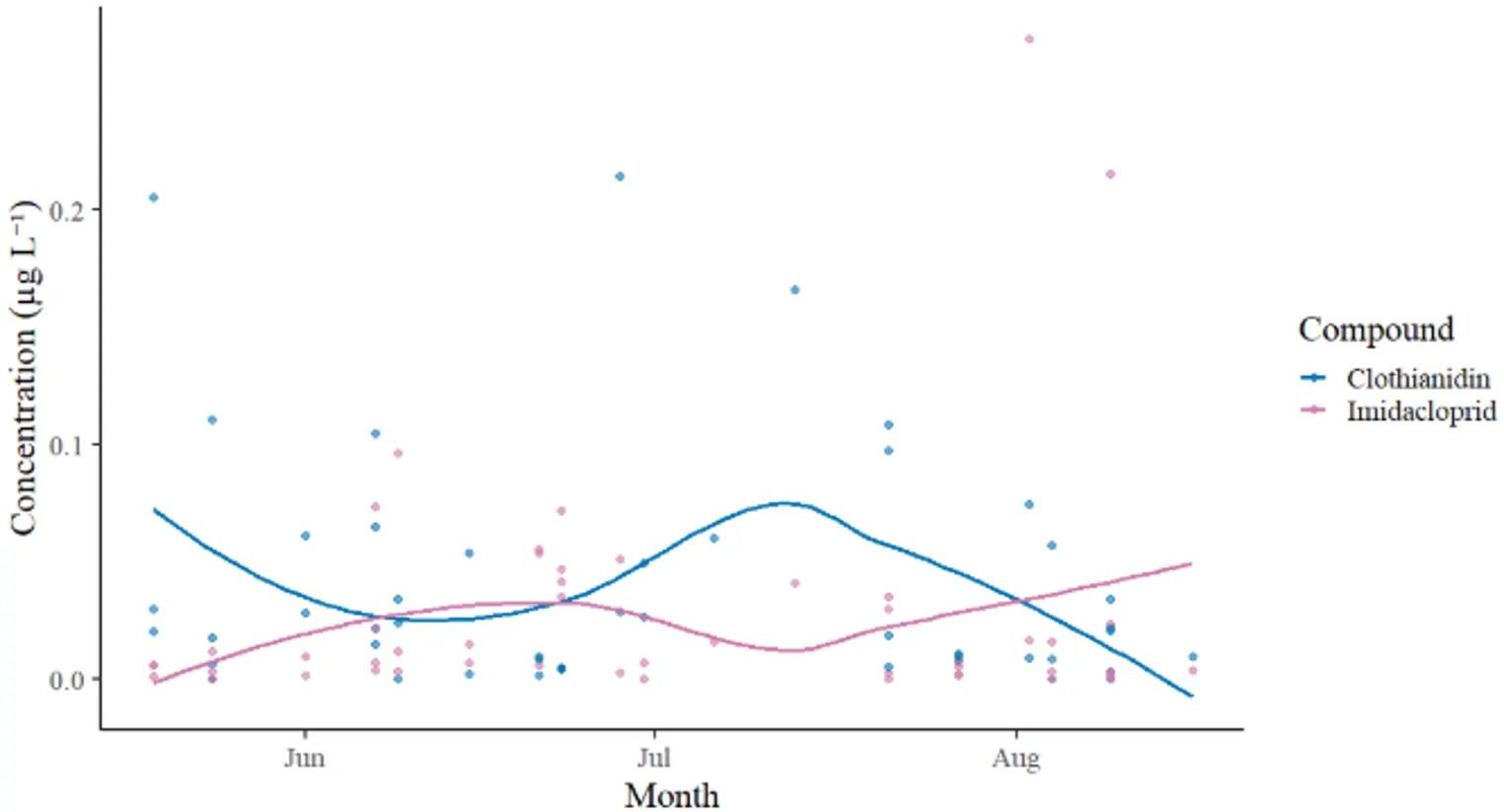
Neonicotinoid concentrations by day of year collected from protected lands (as classified by the Prairie State Conservation Coalition) from 50 samples collected across Illinois in 2021. Points represent observed concentrations and lines represent local polynomial regression fitting

There were 13 sites with clothianidin values above Environmental Protection Agency (EPA) benchmarks (26%) and 22 for imidacloprid (44%), with 50% of surveyed sites having at least one of these chemicals over chronic exposure for freshwater invertebrate benchmark thresholds (Fig. 1). There were no samples that exceeded acute exposure benchmarks. The EPA benchmark for clothianidin is 0.05 µg/L and the highest concentration found at our sites was 0.2145 µg/L. For imidacloprid, the EPA Benchmark is 0.01 µg/L and the highest concentration found for this study was 0.2728 µg/L, over 27-fold higher than the benchmark. Cook County had the highest detection over benchmark for imidacloprid, with four survey sites, all of which exceeded EPA benchmarks. Sites exceeding benchmark for clothianidin were more spatially clustered in central Illinois. We observed a slight increase in concentration for clothianidin in early July and there was not a clear peak for concentration of imidacloprid (Fig. 2). There were six nights where we were unable to obtain insect biomass samples, therefore we used a dataset composed of 94 nights. The range of agricultural land-use ratios was 0.0004 to 0.8141 at the 50 sites sampled. The four lowest agriculture ratio sites were in the Chicagoland area (in Cook or DuPage County).

**Fig. 3:**
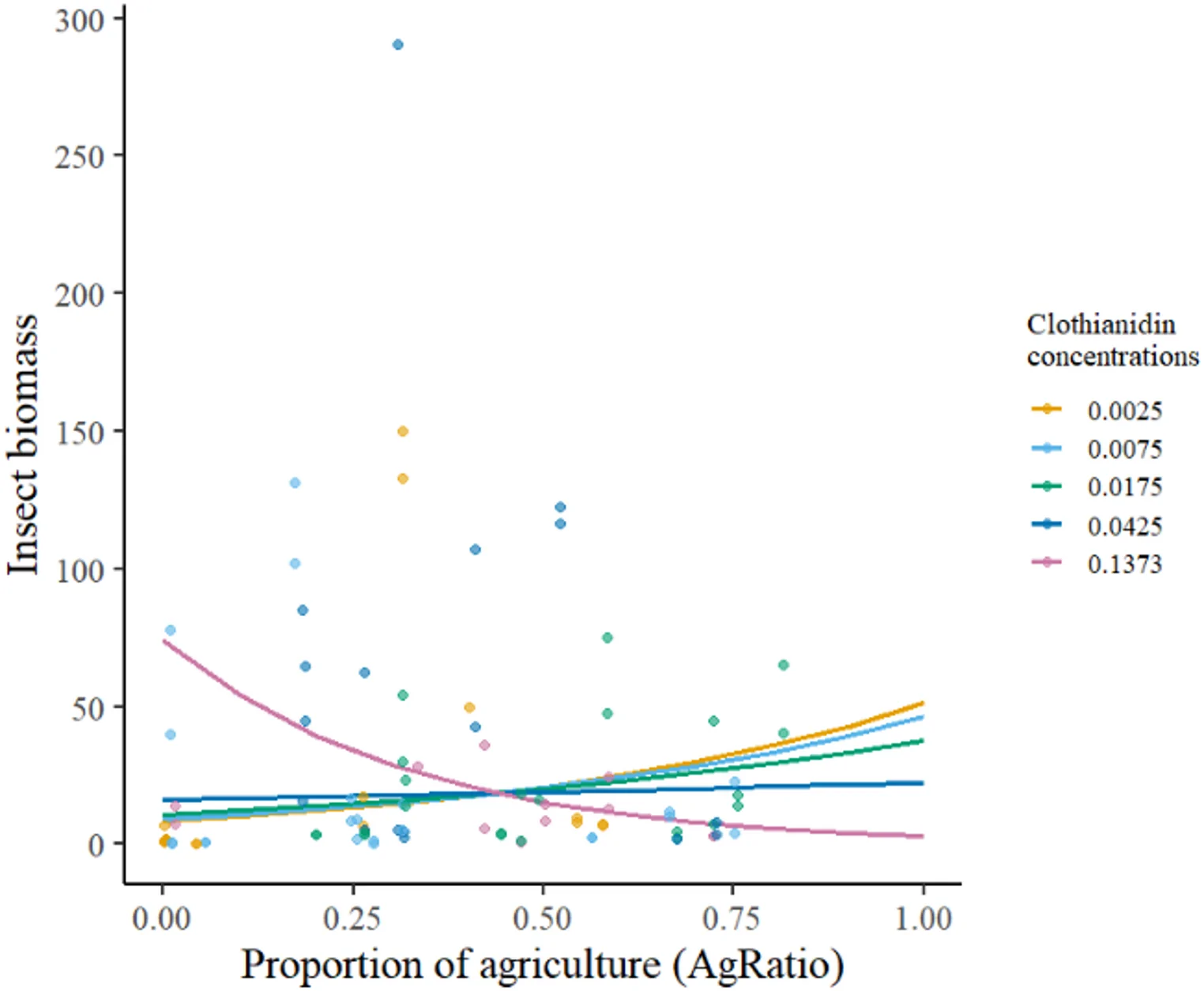
Generalized linear model predicted trendlines for insect biomass by proportion of agriculture and a gradient of clothianidin concentrations (color coding). Points represent observed insect biomass measurements with concentrations binned into nearest clothianidin level for predictions

**Fig. 4:**
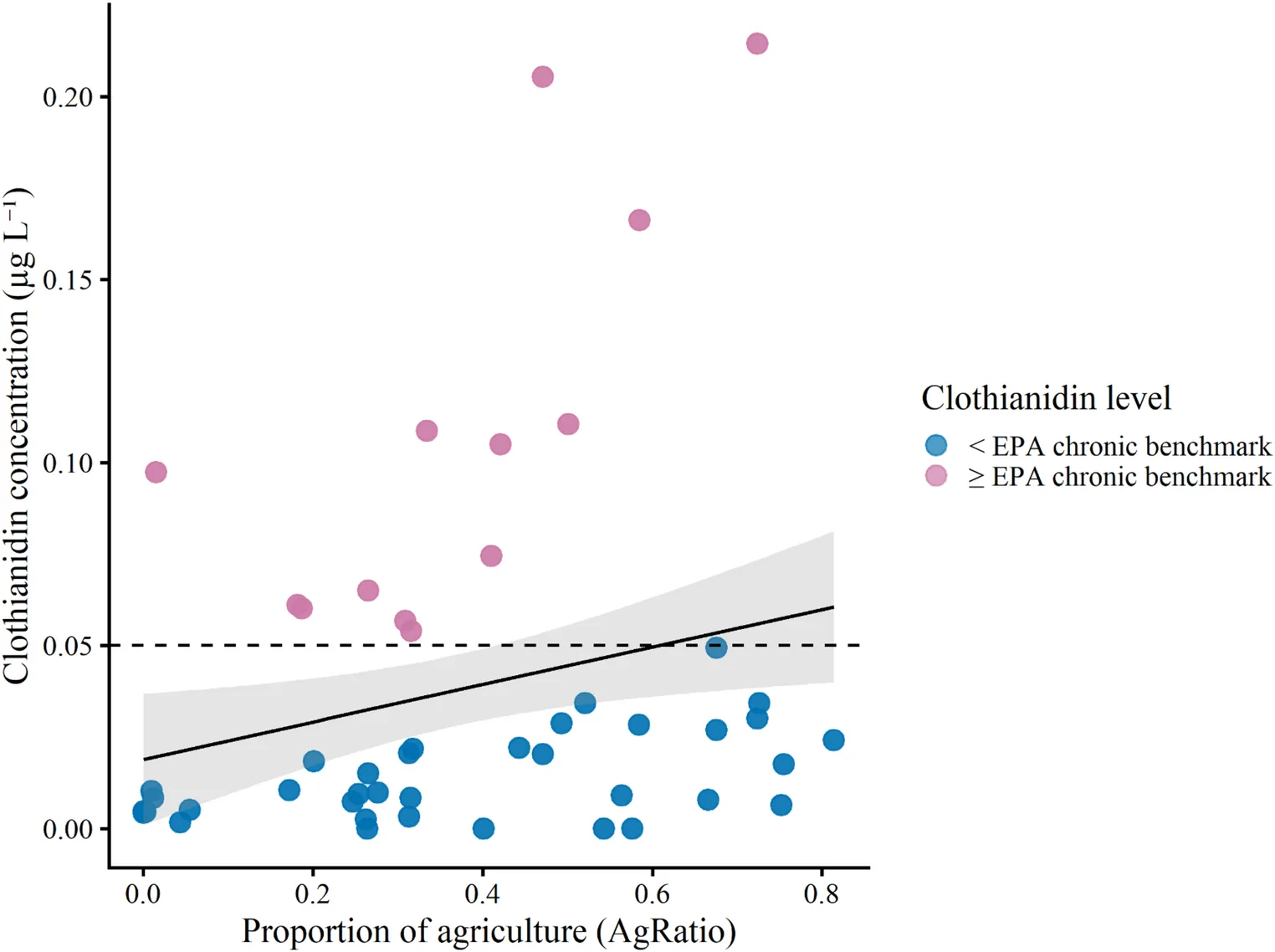
Observed clothianidin concentrations and the proportion of surrounding agricultural land cover (AgRatio). Points are colored according to whether measured concentrations are below or exceed the U.S. EPA freshwater invertebrate chronic benchmark (0.05 µg LL^−1^) indicated by the dashed line

The top model was our global model including clothianidin, total precipitation, ratio of agriculture, nighttime minimum temperature, day of year and latitude (Table 2). However there were three models within ΔAICc of 2, so we used the most parsimonious model which included the ratio of agriculture*clothianidin + latitude. Model fit diagnostics indicated that a Gamma GLM with a log link adequately described variation in insect biomass, with acceptable dispersion (1.42), stable residual behavior, low multicollinearity, and strong support relative to alternative models based on AIC. Clothianidin concentrations had a negative effect on insect biomass at higher ratios of agriculture (Fig. 3). There was limited observed data for sites with low agriculture ratio and high clothianidin, likely making estimation at this intersection skewed (Fig. 4).

**Table 2:** Candidate generalized linear models (using Gamma distribution with a log link) ranked by ΔAICc (Akaike Information Criterion) and including the number of parameters (K), AIC weights (w) and log-likelihood

Model	K	AICc	ΔAICc	AICc w	Log-Likelihood
InsectBio ~ Clothianidin*TotPrecip + AgRatio*Clothianidin + MinTemp*DayofYear + Latitude	11	729.26	0.00	0.42	− 352.02
InsectBio ~ AgRatio*Clothianidin + Latitude	6	730.18	0.92	0.27	− 358.61
InsectBio ~Clothianidin*TotPrecip + AgRatio*Clothianidin + Latitude	8	730.94	1.68	0.18	− 356.63
InsectBio ~ Clothianidin*TotPrecip + AgRatio + Latitude	7	732.75	3.49	0.07	− 358.72
InsectBio ~ Clothianidin*TotPrecip + Latitude	6	734.89	5.63	0.03	− 360.96
InsectBio ~ Latitude	3	735.47	6.21	0.02	− 364.60
InsectBio ~ MinTemp*DayofYear + Latitude	6	737.82	8.56	0.01	− 362.43
InsectBio ~ Clothianidin + AgRatio + Latitude	5	738.03	8.77	0.01	− 363.67

## Discussion

Concentrations of neonicotinoids being higher than EPA benchmarks at 50% of sites is concerning for insects and the ecosystems that depend on them, especially since we exclusively surveyed at non-agricultural sites. There has been widespread documentation of declines in insect populations, likely influenced by many factors, but including the use of pesticides (Goulson 2019; Cardoso et al. 2020). Insects provide ecosystem services in the United States at an estimated $57 billion per year (Losey and Vaughan 2006) making conservation of insect abundance and diversity of critical importance.

The distribution of neonicotinoid contamination in Illinois is likely related to each chemical’s target application. High detection of imidacloprid in Cook County is likely due to this chemical often being used for termites, flea treatment on household pets, and at plant nurseries (Jeschke et al. 2011). Clothianidin is heavily applied to corn and soybean fields, rendering it more prominent in central Illinois, where agriculture is the dominant land cover (Hladik and Kolpin 2015). To compare our results to another nearby state, a study in central Minnesota on Waterfowl Production Areas found detectable levels of clothianidin at 24% of sites and imidacloprid at 7% of sites (Williams and Sweetman 2019).

Given our relatively short survey season, we may have missed higher neonicotinoid levels earlier in the spring or later in the fall. Per Williams and Sweetman (2019), the highest detections were during planting and immediately post-planting. Our results may in fact underestimate peak concentrations if we missed the timing of planting or if concentrations accumulated near the end of the season in fall. Spot sampling likely misses fine scale peaks in contamination (Xing et al. 2013). Similarly, Hladik et al. (2018) also saw slightly higher levels of neonicotinoid concentrations in July and August in the Great Lakes region. Our results may provide some evidence that levels of neonicotinoids in some areas of Illinois exceed the EPA chronic exposure benchmark for longer than just the application peak.

The relationship between clothianidin concentrations, surrounding agriculture and nocturnal insect biomass shows that this chemical is moving into protected lands from nearby agriculture. Efforts to reduce pesticide spread from targeted locations should be implemented, with an emphasis on education to farmers (Reichenberger et al. 2007; Bereswill et al. 2014). We may also include greater consideration of insects in the design and management of protected areas (Chowdhury et al. 2023). As of 2022, the EPA determined that clothianidin is likely to adversely affect 67% of listed endangered species and imidacloprid to affect 79% of species (USEPA 2022). Our study provides additional evidence that even in the protected lands of Illinois, these contaminants are persistently present, often exceed EPA benchmarks and are likely impacting nocturnal insect populations.
